# In this month’s Bulletin 

**DOI:** 10.2471/BLT.23.000623

**Published:** 2023-06-01

In the editorial section, Giorgio Cometto et al. (362) explain provisions of WHO’s 2023 health workforce support and safeguards list. Katherine Pettus et al. (363) argue that a future pandemic treaty should include provisions for palliative care. Sameer Pujari et al. (364) advise that cautious optimism is warranted for artificial intelligence for global health.

Tatum Anderson (367 – 368) reports on projects to eliminate mercury in skin lightening products in Gabon, Jamaica and Sri Lanka. John Rex talks to Gary Humphreys (369 – 370) about the challenges faced in developing new antibiotics and bringing them to market. 

## China 

### Cervical cancer prevention

Xingce Zhu et al. (381 – 390) describe how artificial intelligence was used in a screening programme.

## Kenya, Malawi, Nigeria, Sierra Leone, Uganda, United Republic of Tanzania, Zambia.

### Antimicrobial stewardship

Nduta Kamere et al. (403 – 411) analyse supply chain factors

## Central African Republic

### Meeting coverage targets

Adidja Amani et al. (431 – 436) describe the roll-out of COVID-19 vaccination.

## Zambia

### Community treatment of severe malaria 

Cathy Green et al. (371 – 380) study the use of rectal artesunate.

## Global 

### Kangaroo mother care 

Barsha Gadapani Pathak et al. (391– 402) review the evidence of effects on maternal and paternal health.

### Refurbished medical devices

Shatrunajay Shukla et al. (412 – 417) discuss how to improve regulatory practices

### Occupational risk factors

Frank Pega et al. (418 – 430) present a new global indicator for workers’ health.

